# The Herlyn-Werner-Wunderlich (HWW) syndrome – A case report with radiological review

**DOI:** 10.1016/j.radcr.2022.02.017

**Published:** 2022-03-02

**Authors:** Abdul Malik Hayat, Khalid Rehman Yousaf, Saman Chaudhary, Sohaib Amjad

**Affiliations:** a Department of Radiology, Kabul University of Medical Sciences, Kabul, Afghanistan; b Department of Radiology, Sir Ganga Ram Hospital, Lahore, Pakistan

**Keywords:** Dysmenorrhea, Hematometrocolpos, Mullerian ducts, Didelphys, Renal agenesis, Infertility

## Abstract

Herlyn-Werner-Wunderlich (HWW) syndrome is a rare congenital anomaly of female urogenital tract involving combined mullerian duct anomalies and mesonephric duct malformation characterized by uterus didelphys, obstructed hemi-vagina and ipsilateral renal agenesis which is also known as OHVIRA syndrome. It can be classified based on a completely or incompletely obstructed hemivagina. It presents soon after menarche or shows delayed presentation depending on the type. The most common presentation is lower abdominal pain, dysmenorrhea, and abdominal mass in the lower abdomen secondary to hematometra or hematocolpos. We present a 15-year-old unmarried patient with an unusual case of OHVIRA syndrome suffering from dysmenorrhea and painful mass in suprapubic region. We described the role of imaging modalities in diagnosis of the Herlyn-Werner Wunderlich syndrome with a review of literature. On USG and MRI, she had right renal agenesis with compensatory hypertrophy of the left kidney, didelphic uterus with an obstructed hemi-vagina on right side which led to marked distention of ipsilateral cervix and proximal vagina in the form of hematometrocolpos. OHVIRA syndrome can present early or late, depending on the type. In patients with uterine and vaginal abnormalities, a work-up for associated renal anomalies should be performed. The choice imaging modalities for the diagnosis of OHVIRA syndrome are ultrasound and MRI. Knowing the imaging findings of this rare condition is crucial for early diagnosis in order to prevent complications which may lead to endometriosis and infertility.

## Introduction

Herlyn-Werner-Wunderlich (HWW) syndrome is a rare female urogenital anomaly characterized by uterus didelphys, unilateral obstructed hemivagina and ipsilateral renal agenesis. It is also known as OHVIRA which represents a diagnostic dilemma because of the regular menstruation and nonspecific abdominal pain. It resulted from the combination of mesonephric and mullerian ducts anomaly [1,2]. The exact incidence of this syndrome is unknown [3], but its estimated occurrence is 0.1%-3.8% [1].

Herlyn-Werner syndrome was initially described in 1971 by Herlyn and Werner [4]. In 1976, Wunderlich described an association of right renal aplasia with a bicornuate uterus and simple vagina in the presence of an isolated hematocolpos [5].

It is presented in post menarch young adults with irregular menses, lower abdominal pain and pelvic mass [6], which in most of the time is not ruled out due to its rarity.

Early detection and septoplasty (surgical resection of obstructing vaginal septum) will relieve pain and prevent further complications such as endometriosis and infertility [7].

The unique feature of our case is right sided hematometrocolpos with right sided atresia of cervix and vagina along with right renal agenesis.

## Case report

A 15 years old unmarried girl was presented with lower abdominal pain accompanying her menstral cycle and occational vomiting. Her menarch started at 13 years of age with regular menses.

**Fig. 1:**
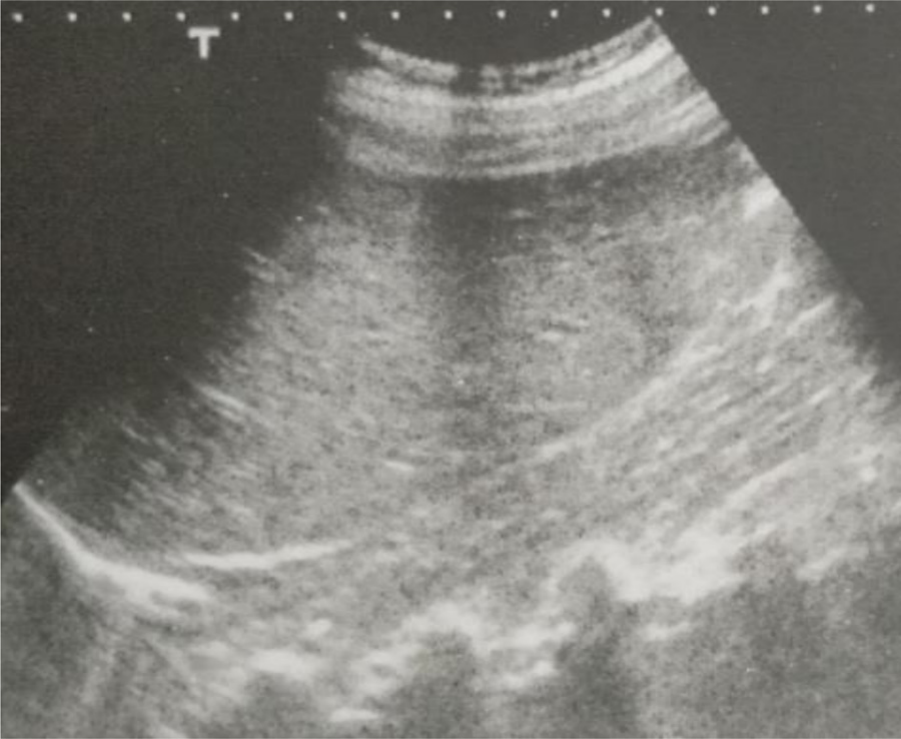
Ultrasound of the abdomen revealed absence of the right kidney.

**Fig. 2:**
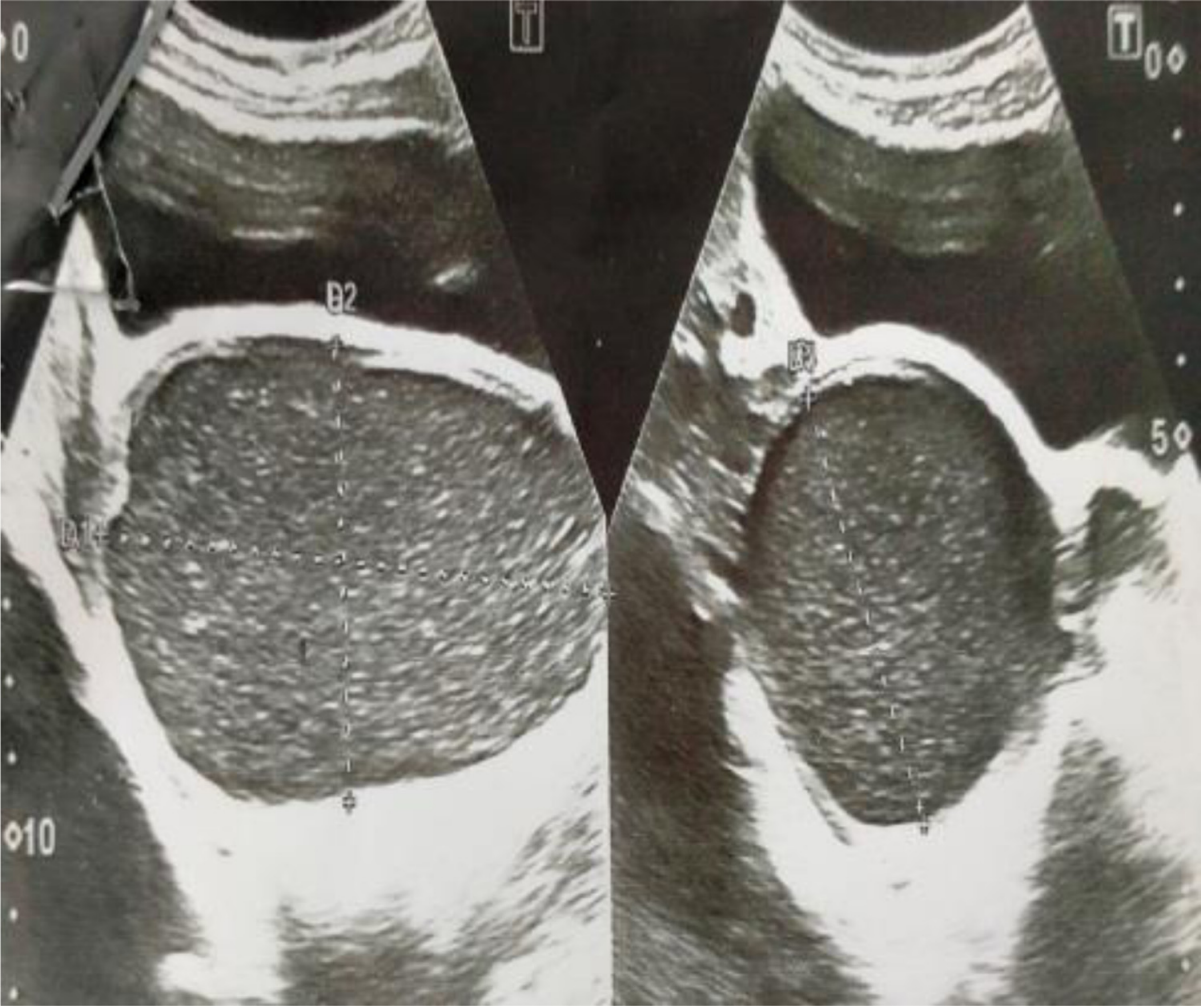
USG of pelvis demonstrates cystic dilatation of cervix having internal internal echoes.

Ultrasound of the abdomen and pelvis revealed absence of the right kidney (Fig. 1). The left kidney showed compensatory hypertrophy and measured 12.3 × 5.8 cm. Uterus didelphys was noted. The right uterine cavity and cervical canal showed a hypoechoic collection with multiple internal echoes suggestive of hematometrocolpos (Fig. 2). The left uterine horn and cervical canal appeared normal. No gross adnexal pathology seen. Both ovaries visualized normally.

**Fig. 3:**
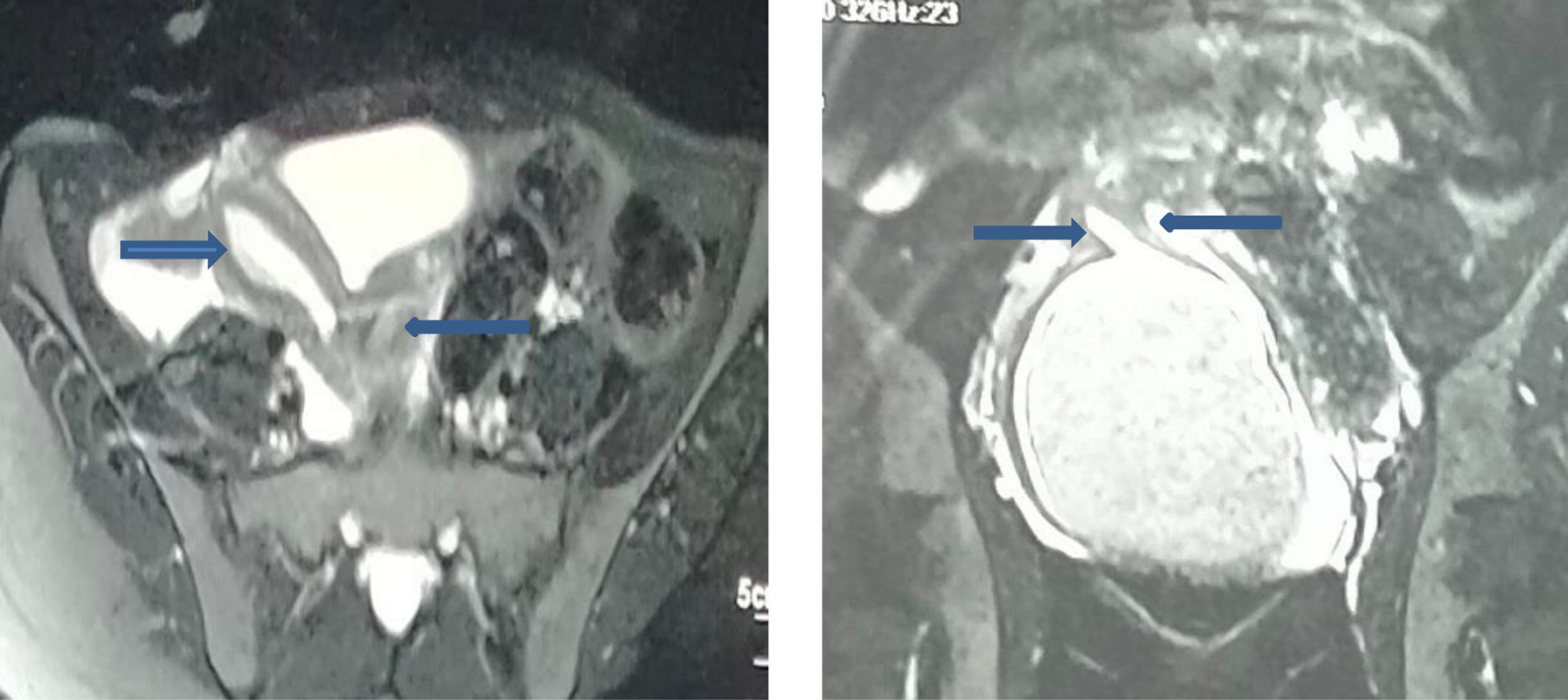
MRI Pelvis demonstrates, (A) uterus didelphys, (B) hematometrocolpos and 2 endometrial cavities.

**Fig. 4:**
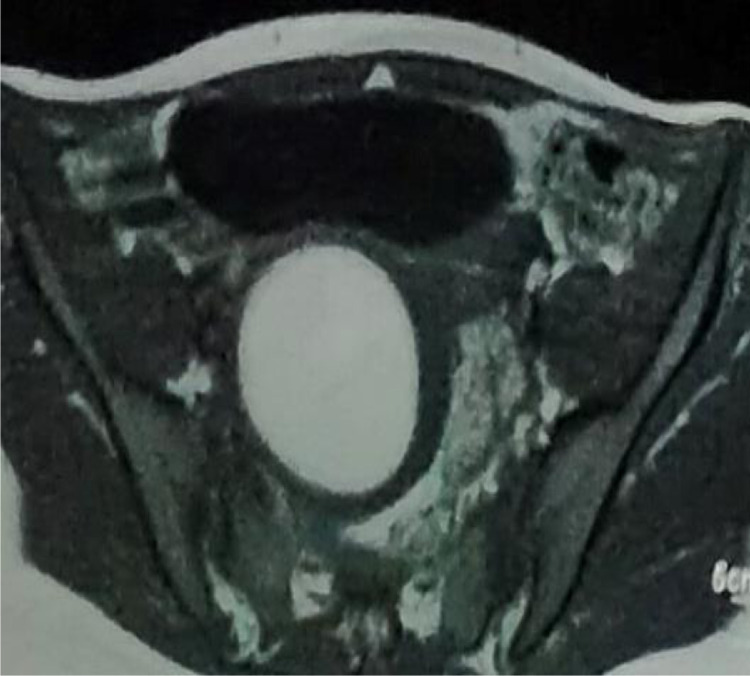
Axial MRI pelvis demonstrates hematocolpos with compressed leftt hemivagina.

MRI pelvis revealed uterus didelphys with distended right sided cervical and vaginal canal with fluid following signals of sub-acute hemorrhage having volume of 6.2 × 8.1 × 14.1 cm in APxTRxCC dimensions respectively (Fig. 3). The lower end of fluid collection in the cervix showed inferior convexity suggestive of obstruction at the level of the cervix or proximal hemivagina. Right endometrial cavity measures 13 mm in thickness and contains streak of fluid in it. Left endometrial cavity measures 6mm in thickness and is displaced and compressed by the distended obstructed right hemivagina Fig. 4. Left cervical is also effaced towards left side as well. Both ovaries are normal.

**Fig. 5:**
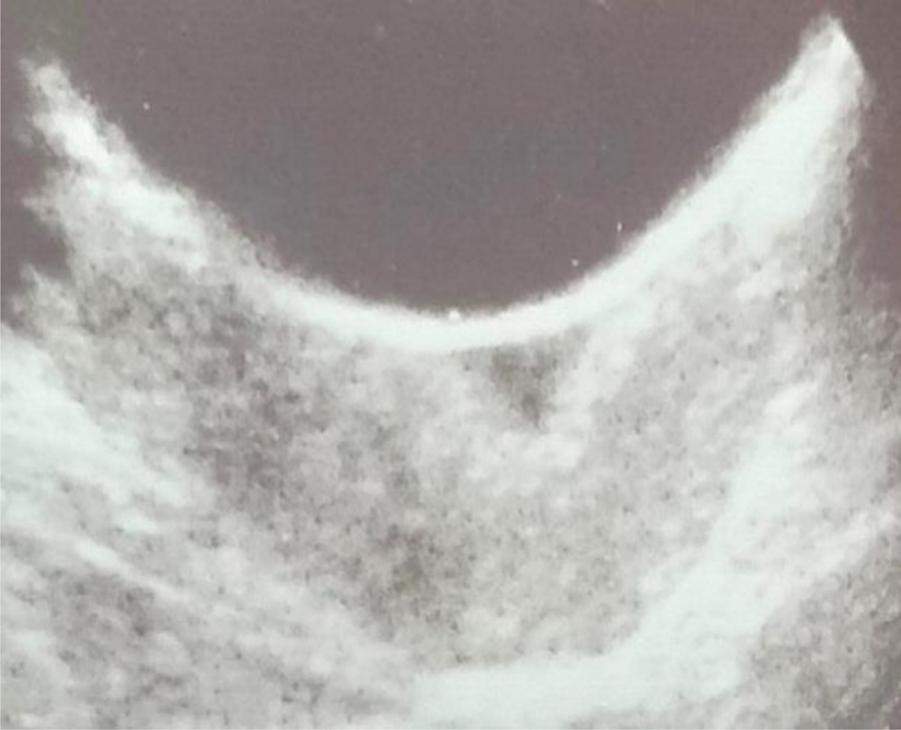
Post operative USG demonstrates no collection.

These findings of uterus didelphys with right hematometrocolpos and right renal agenesis are suggestive of the Herlyn-Werner-Wunderlich syndrome [classification 1.2]. Obstruction was at the level of the cervix or proximal hemi-vagina. Septoplastywas performed. Post operative ultrasound showed no collection, however there was streak of fluid in right endometrial cavity (Fig. 5).

## Discussion

A useful classification of mullerian duct anomaly was described by Buttram and Gibbons [7]. Uterine didelphys results from non-development, defective fusion of the müllerian ducts or defects in regression of the septum during foetal development [8]. It is classified as Class 1,2 MDA and is accounted for 5% of MDAs [4,5]. It is associated by mesonephric duct anomaly. The Wolffian duct is responsible for the formation of the kidney. Accordingly, in its absence on 1 side, the kidney and ureter of the same side will fail to fuse [4,5].

Wolffian ducts are inductor elements for adequate fusion of the Mullerian ducts, therefore in the absence of the mesonephric (Wolffian) duct, the paramesonephric (müllerian) duct is displaced laterally and fails to adequately fuse with the urogenital sinus, leading to the formation of a blind sac, imperforated or obstructed hemi-vagina [3]. The distal part of vagina which arises from the urogenital sinus is not affected and develops normally.

Lan Zhu et al., suggested a new classification for the syndrome based on the presence of a completely and incompletely obstructed vaginal septum [9]. This classification assists in earlier diagnosis and treatment. The syndrome is classified type 1 (completely obstructed hemivagina) and type 2 (incompletely obstructed hemivagina). The clinical findings in these 2 types are different (Table 1).

**Table 1:** Lan Zhu et al. new classification of HWW syndrome.

Renal agenesis is ipsilateral to the dilated uterine cavity. The right side is affected twice more frequently than the left side [10,11]. Other associated anomalies include renal dysplasia, duplication of the kidneys and ureters, ectopic ureter, high-riding aortic bifurcation, IVC duplication, intestinal malrotation and ovarian malposition [8].

Patients with OHVIRA syndrome are usually asymptomatic until puberty. Diagnosis is usually made soon after menarche and the presenting symptoms are pelvic pain, dysmenorrhea, and pelvic mass [12,13]. If not treated, complications leading to infertility, endometriosis, pelvic adhesions, and pyosalpinx or pyocolpos which may present in the late phase with a high miscarriage rate [12]. Rare complication of adenocarcinoma and clear cell carcinoma of the obstructed side of the uterine cervix and of the vagina have been also documented [8].

## Diagnosis

The choice imaging modalities for the diagnosis of OHVIRA syndrome are ultrasound and MRI, both of which have an added advantage of being non-invasive [1], [2], [3], [4], [5].

Ultrasound may reveal uterine didelphys and pelvic fluid collection with low level internal echoes, contiguous with the hemato/pyocolpos. Due to retrograde menstruation, features of endometriosis in form of well defined, unilocular or multilocular, predominantly cystic masses containing diffuse, homogeneous, low level internal echoes may also be seen [14].

MRI plays an important role in characterizing the didelphic uterus, obstructed hemivagina, and ipsilateral renal agenesis [1,10]. MRI findings of OHVIRA syndrome are characterized by iso/high T1W signal and high T2W signal that indicate pelvic fluid collection is contiguous with the endocervix along with didelphic uterus and an absent kidney on the affected side.

MRI is far better than ultrasound for characterizing anatomical relationships due to its multiplanar capabilities and larger field of view [2]. However, the gold standard for diagnosis of OHVIRA syndrom is laproscopy, which has the benefit of performing therapeutic drainage of hematometrocolpos, vaginal septotomy and marsupialization [15]. Treatment usually involves surgery in the form of septoplasty which helps in relieving obstruction [16]. Surgical intervention also decreases the chances of pelvic endometriosis due to retrograde menstruation. About 87% of patients go on to have a successful pregnancy; however, 23% of patients carry the risk of subsequent abortion [17].

The rarity of OHVIRA syndrome complicates its diagnosis, and hence clinicians and radiologists should consider MDAs among the differential diagnosis in young female patients presenting with abdominal symptoms, especially when associated with renal agenesis. Understanding the imaging findings is critical for early diagnosis in an attempt to prevent complications.

## Conclusions

HWW syndrome has variable onset of presentation. It is necessary to perform work up for associated renal anomalies in patients with uterine and vaginal abnormalities. USG has advantage of low cost & real time imaging. MRI which has multiplanar imaging capability with no radiation hazard, is considered gold standard for the diagnosis. It can also detect associated renal agenesis and complications like endometriosis. It helps clinicians for planning, staging, assessing risk-benefit ratio of different treatment approaches. Early intervention is needed to reduce risk of endometriosis and infertility.

## Patient consent

Patient consent has been obtained.
